# The impact of the COVID-19 pandemic on palliative care practice: A survey of clinical oncologists

**DOI:** 10.3389/fpubh.2022.1020937

**Published:** 2022-11-21

**Authors:** Yu Wang, Yilin Huang, Xiangyu Ma, Dilinaer Wusiman, Xinqing Zhang, Nan Bi

**Affiliations:** ^1^Department of Radiation Oncology, National Cancer Center/National Clinical Research Center for Cancer/Cancer Hospital, Peking Union Medical College and Chinese Academy of Medical Science, Beijing, China; ^2^State Key Laboratory of Cardiovascular Disease, Division of Prevention and Community Health, National Center for Cardiovascular Disease, National Clinical Research Center of Cardiovascular Disease, Fuwai Hospital, Peking Union Medical College and Chinese Academy of Medical Sciences, Beijing, China; ^3^Department of Head and Neck Surgery, National Cancer Center/National Clinical Research Center for Cancer/Cancer Hospital, Peking Union Medical College and Chinese Academy of Medical Sciences, Beijing, China; ^4^School of Humanities and Social Sciences, Peking Union Medical College, Beijing, China

**Keywords:** palliative care, COVID-19, pandemic, oncologist, survey

## Abstract

**Background:**

Palliative care is an essential intervention to improve the quality of life for patients with cancer, whereas the ongoing COVID-19 pandemic poses a challenge to supportive and palliative care providers. This survey aims to explore the current status of palliative care practice for cancer and the influence of COVID-19, from the perspective of oncologists.

**Methods:**

The semi-structure electronic questionnaire was designed. Mixed-mode surveys including electronic questionnaires, face-to-face interactions, and telephone interviews were adopted according to the willingness of respondents. Face-to-face and telephone interviews were based on same questions in the online questionnaire. Participants working in cancer-related departments with frontline palliative care experience during the COVID-19 outbreak were included. Surveys covered experiences and perspectives regarding the impact of COVID-19 on clinical work, personal lives, and palliative care practice. Suggestions on coping strategies were further proposed and qualitatively analyzed.

**Results:**

Thirty-seven oncologists participated in this study from September 2021 to January 2022. The majority of them believed COVID-19 significantly and negatively affected their clinical work routines (75.7%), personal daily lives (67.6%), and palliative care practice (64.9%). Most specialists considered that currently the palliative care system remained underdeveloped (73.0%), and other factors besides COVID-19 were associated with this situation (78.4%). Seventeen participants further made suggestions on how to promote palliative care during COVID-19, and three themes emerged through the qualitative analysis: (1) Remote or online service (88.2%); (2) Publicity, education, or shared decision-making for patients (29.4%); (3) Guidelines, training, or programs for care providers (23.6%).

**Conclusion:**

Oncologists consider that COVID-19 has an adverse impact on their palliative care practice and daily routine. In addition to COVID-19, other factors affecting palliative care should not be neglected. Corresponding measures are warranted to encourage palliative care practice during COVID-19.

## Introduction

In December 2019, coronavirus diseases 2019 (COVID-19), caused by severe acute respiratory syndrome coronavirus 2 (SARS-CoV-2), emerged in China. Subsequently, COVID-19 has rapidly become a severe pandemic and significantly impacted various clinical practices (1, 2). Although the global mortality rate estimated by the World Health Organization (WHO) was 3.4%, mortality and morbidity rates tend to be higher among older people and cancer patients (1, 3). The outbreak of the COVID-19 pandemic results in a global shortage of healthcare resources, presumably including supportive and palliative care resources and applications, especially for the large number of patients with cancer (4). At present, cancer remains the leading cause of worldwide medical burden and brings tremendous physical and mental stress on patients and their families (5). Palliative care is an essential component of the cancer comprehensive treatment, aiming to alleviate the suffering and improve the quality of life (6). Given that COVID-19 is expected to surpass our capacity to provide supportive and palliative care to all patients, which poses a unique challenge to healthcare teams of rationing care during pandemic when resources are scarce (4), in-depth investigations on the specific impact of the ongoing COVID-19 pandemic on the clinical practice of palliative care for cancer patients as well as effective coping strategies are necessary.

The two-sides of practice indicates that one side is the recipient of care and the other side is for the care providers. Thus, both care practitioners and patients play an important role in the practice of palliative care. With early studies suggesting that cancer patients are particularly susceptible to COVID-19, the current pandemic is forcing oncology professionals to explore and practice more (3, 7, 8). Meanwhile, not only vulnerable patients affected by COVID-19 should be concerned, but also palliative care practitioners. The experiences, perspectives and thoughts of care providers during COVID-19 are of great importance and value, even though sometimes we may mainly focus on patients and neglect that doctors are passive sufferers of the COVID-19 pandemic as well. In fact, the health care workers struggling to cope with the current situation face more stress and anxiety, due to the heavier medical burden under COVID-19 and their overwork (9). The fact that the medical staff come so close to the disease puts their mental health at a higher risk than the general population (10). Recently, an increasing prevalence of mental health symptoms has been reported among physicians who had direct contact with infected patients (11, 12). With the explosive growth of the number of diagnosed COVID-19 cases, the stress, anxiety, depression, and feelings of negativity became more and more common in Chinese medical workers (13, 14).

With the increasing difficulty to provide palliative care during the COVID-19 pandemic, the wide emphasis on the experiences and viewpoints of palliative care providers, as well as the urgent need for useful coping strategies to better tackle the influence of COVID-19 on palliative care practice, this survey was conducted in order to shed light on these issues. We not only explored the impact of the COVID-19 pandemic on the clinical practice of supportive and palliative care for patients with cancer, from the perspective of oncologists, but also proposed some useful countermeasures to promote and encourage the clinical practice of palliative care during COVID-19.

## Materials and methods

### Study design

We designed a semi-structured electronic questionnaire to elicit the perspectives of clinical oncologists through both quantitative and open-ended qualitative questions. Mixed-mode surveys including online electronic questionnaires, face-to-face interactions, and telephone interviews were adopted based on the willingness of respondents. If they agreed to receive face-to-face or telephone surveys, they would be individually asked the same questions in the online electronic questionnaire in person, and the information was collected. After obtaining permission from respondents in face-to-face and telephone surveys, all interviews were digitally recorded and transcribed verbatim to assure accuracy. If participants chose to answer the electronic questionnaire, they would complete a self-administered anonymous web-based questionnaire in both Chinese and English. The Independent Ethics Committee of National Cancer Center approved this research. Informed consent was obtained from all individual participants included in the study. They can access the online Participant Information Consent Form via a secure web link and complete it using mobile phones or computers. After the completion of survey, participants could receive an e-card gift and a thank-you note *via* email, if they were willing to provide their email addresses and some other personal information only for this purpose.

### Participants and study setting

The study was conducted from September 2021 to January 2022 in China. In the approximately two-year pandemic background, this survey was primarily in the setting where palliative care was provided for cancer patients during the COVID-19 period, including medical centers, hospitals, nursing homes, palliative care institutions, community healthcare centers, etc.

Eligible participants were those over 18 years of age currently working in cancer-related departments, such as the department of medical oncology, radiation oncology and surgical oncology, etc, in medical establishments or other sites that provide palliative care practice. Among them, oncologists who had clinical front-line working experiences during the COVID-19 pandemic (from December 2019 to the date participating in the survey), possessed and were able to use online electronic mobile devices autonomously, and proficiently mastered Chinese or English language, were finally included.

### Survey content

The questionnaire survey or interview outline covered: (1) Demographic characteristics: Age, gender, country, educational attainment, workplace and currently working department, supportive and palliative care training experience, the primary place of palliative care practice.

(2) Subjective perceptions regarding the influence of the COVID-19 pandemic: Do you think the COVID-19 pandemic has a significant impact on your clinical work in oncology? Yes, mainly negative impacts. / Yes, mainly positive impacts. / No. / Other. Do you think the COVID-19 pandemic has a significant impact on your personal life or daily routine? Yes, mainly negative impacts. / Yes, mainly positive impacts. / No. / Other. Do you think the COVID-19 pandemic has a significant impact on clinical practice of palliative care for cancer patients? Yes, mainly negative impacts. / Yes, mainly positive impacts. / No. / Other.

(3) Subjective perspectives regarding the status quo of palliative care: Do you agree that the current supportive and palliative care system in your working environment is adequate or fully developed? Yes. / No. / I am not sure. / Other. Do you agree that other factors, except for the influence of COVID-19, are associated with the current status of palliative care practice system? Yes. / No. / I am not sure. / Other.

(4) Suggestions and advice: Do you have any suggestions to improve the clinical practice of palliative care during the COVID-19 pandemic? If yes, please give your precious and specific advice.

### Data collection and analysis

Data was collected from the electronic questionnaire surveys and interviews on oncologists in China from September 2021 to January 2022. Demographics and subjective perspectives of respondents were quantitatively summarized mainly using descriptive statistics. Personal suggestions of free-text narrative responses were qualitatively analyzed through inductive thematic analyses. All data were translated into English before analysis. All face-to-face and telephone interview surveys were digitally recorded and transcribed verbatim by two researchers (W.Y. and X.M.) together to assure maximum accuracy. Two investigators (W.Y. and D.W.), without previous knowledge of the participants and not involved in the distribution of questionnaires, independently collected and analyzed the questionnaire content, and they further compared and verified their research results. Any discrepancies between two researchers (W.Y. and D.W.), especially in summarizing countermeasures proposed by participants, were solved by consulting senior investigators (X.Z. and N.B.).

## Results

Thirty-seven eligible clinical oncologists participated in this study. Among them, 32 (86.5%) were surveyed by online questionnaires, 4 (10.8%) by face-to-face interviews, and 1 (2.7%) by a telephone interview. The baseline demographic characteristics were presented in Table 1. All 37 participants were from China, including 29 (78.4%) males. The median age was 40 (22–56). A large proportion of them had doctoral degrees (67.6%) and worked in urban areas (81.1%). One-third (32.4%) participants were from the department of radiation oncology, 24.3% from medical oncology, and 16.2% from surgical oncology. Meanwhile, clinicians were more likely to practice palliative care in medical centers or hospitals (70.3%) than in the community or elsewhere (29.7%) in China. However, only 10.8% of them had obtained the accredited professional training certification in palliative medicine, and most were with non-accredited training experience (56.8%).

**Table 1:** Demographic characteristics of survey participants (*n* = 37).

	**Participants *n* (%)**
**Age (median, range), years**	**40 (25–56)**
**Gender**	
Male	29 (78.4)
Female	8 (21.6)
**Country**	
China	37 (100)
Other	0
**Educational attainment**	
MD or PhD	25 (67.6)
Master degree	10 (27.0)
Undergraduate degree	2 (5.4)
**Urban or rural workplace**	
Urban	30 (81.1)
Rural or other	7 (18.9)
**Currently working department**	
Radiation oncology	12 (32.4)
Medical oncology	9 (24.3)
Surgical oncology	6 (16.2)
Other	10 (27.0)
**Training in palliative medicine**	
Accredited training	4 (10.8)
Non-accredited training	21 (56.8)
No training or other condition	12 (32.4)
**Place of palliative care practice**	
Medical center/hospital	26 (70.3)
Community or other	11 (29.7)

**Figure 1:**
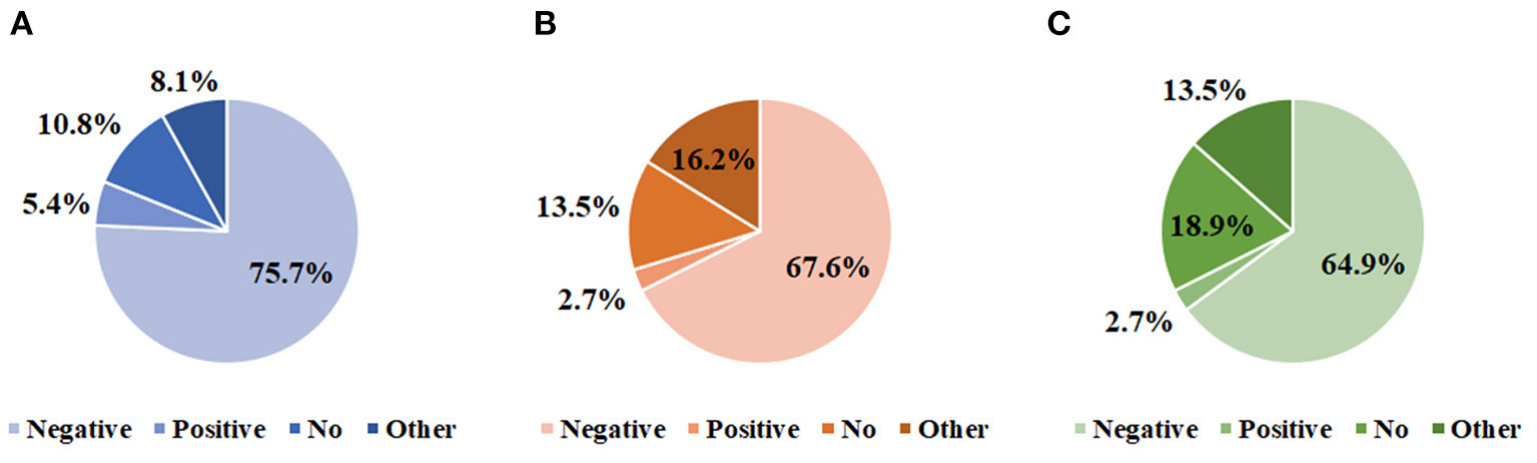
Experiences and perspective of oncologists regarding the impact of COVID-19. The extent to which COVID-19 affected clinical work routine in oncology **(A)**, personal daily lives **(B)**, and palliative care practice **(C)**.

In terms of subjective perceptions and opinions with respect to the impact of COVID-19, the majority of participants agreed that the COVID-19 pandemic had a significantly negative effect on their clinical work in the cancer field (75.7%, Figure 1A), as well as their daily routines or personal lives (67.6%, Figure 1B). In addition, as many as of 64.9% specialists considered that the clinical practice of palliative care for cancer patients had been significantly and negatively affected (Figure 1C). Moreover, 2 (5.4%) oncologists believed that the current supportive and palliative care system was fully developed in China, while 73.0% of them deemed that it remained underdeveloped. It was quite common for them to agree that many other factors besides COVID-19 were associated with this present status (78.4%), but 2.7% of participants disagreed with that.

Furthermore, a total of 17 specialized physicians proposed their suggestions on how to tackle the adverse influence of COVID-19 on palliative care practice. The qualitative analysis resulted in the following three themes: (1) Remote or online service (88.2%); (2) Publicity, education, or shared decision-making for patients (29.4%); (3) Guidelines, training, or programs for care providers (23.6%). We reported some suggestions made by participants (P) in Box 1.

Box 1 **Quotes per theme.**

“*Theme 1. Offering remote or online service”*

Offering more online guidance on palliative care for cancer patients and their families

“*Before the COVID-19 epidemic, there were more cancer patients from other provinces in our hospital, but due to the restrictions of the epidemic, the access and follow-up of these patients were very limited. Adding more online consultations may help them” #P25*

Promoting remote multidisciplinary cooperation

“*Multidisciplinary cooperation members cannot meet on-site due to epidemic restrictions, but the mechanism of online MDT (multidisciplinary treatment) is not mature enough and needs to be improved urgently” #P34*

“*Invite the professional palliative medicine team to join the multidisciplinary consultation” #P9*

Strengthening home-based palliative care

“*…palliative care at home for cancer patients should be given a higher priority, and access to remote medical guidance is less clear for physicians as well as patients” #P20*

“*The trend of shifting palliative care settings from hospital to home has been accelerated by COVID-19” #P37*

Using more modern technology in palliative care

“*…mobile phones and WeChat could become important tools to provide remote palliative care guidance during this COVID-19 period. It would be better to have some platforms like WeChat public account for telemedicine that do not involve doctors' personal privacy” #P32*

*Theme 2. Increasing the publicity, education, or shared decision-making for patients*

Encouraging shared decision-making

“*The situation of patients who need palliative care is often complex, especially end-of-life patients, and it could be more helpful and effective if patients and doctors share the decision-making process” #P35*

Increasing the publicity and education of palliative medicine

“*Increase palliative care education, positive publicity, and concept shaping, the actual potential demand in China is huge” #P19*

*Theme 3. More guidelines, training, or programs for care providers*

Standardizing palliative medicine training for medical workers

“*Palliative care providers working in different sites, different regions, and different fields will need more specialized training” #P11*

“*Most care providers actually do not specialize in this. They might lack relevant professional experience. More professional training in palliative medicine might be helpful” # P36*

Enhancing personalized palliative care programs

“*During COVID-19, government support may be more helpful, such as more palliative care programs and financial investments” #P5*

“*…promoting projects about personalized palliative care practices could be of great benefit, given that each doctor's situation (under the COVID-19 pandemic) is different” #P37*

Updating more information on COVID-19 in palliative care guidelines

“*The prevention and control of the COVID-19 in China is becoming more and more regular. Information and guidelines on palliative care also should be updated accordingly” #P35*

**Figure 2:**
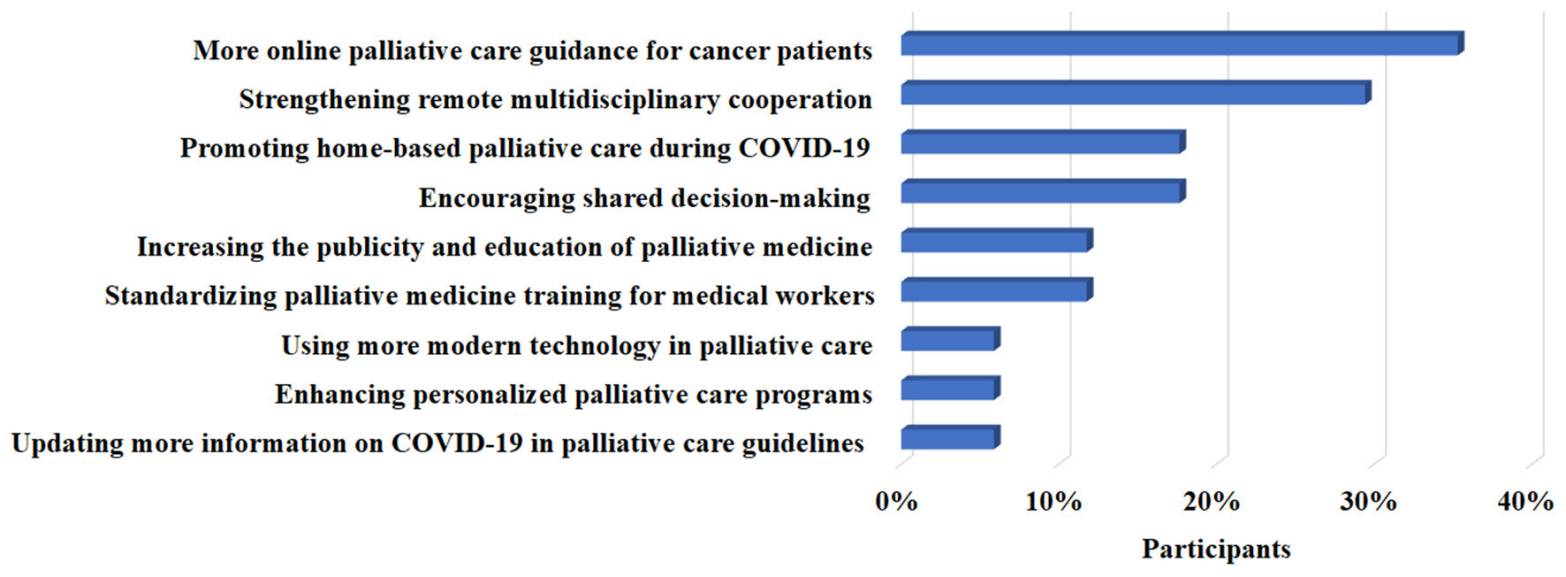
Personal suggestions on developing palliative care under the COVID-19 pandemic.

A total of 17 participants (including P2, P5, P9, P11, P16, P19, P20, P24, P25, P27, P29, P31, P32, P33, P34, P36, and P37) answered the free-text questions and gave their narrative suggestions on coping strategies. Among them, P32, P33, P34, P36, and P37 were interviewed face to face or by telephone. These suggestions were also summarized in Figure 2, which included offering more online guidance on palliative care for cancer patients and their families (35.3%), promoting remote multidisciplinary cooperation (29.4%), strengthening home-based palliative care (17.6%), encouraging shared decision-making during the practice (17.6%), increasing the publicity and education of palliative medicine (11.8%), standardizing palliative care training for medical workers (11.8%), using modern technology in supportive care practice (5.9%), enhancing personalized palliative care programs (5.9%), and updating palliative care guidelines with more information on COVID-19 (5.9%).

## Discussion

It is known that the integration of team-based, timely and targeted supportive and palliative care into standard oncology care for all patients with cancer is of great significance (15). The characteristics of palliative care mainly lie in the team-based care, allowing the interdisciplinary members to address comprehensively the multi-dimensional care needs of patients and their caregivers; the timely intervention, becoming preventative care to minimize crises at the end-of-life; and the targeted treatment, referring to the identification of the patient most likely to benefit from a specialized palliative treatment (15). Nevertheless, palliative care services are under-resourced at the best of times (16). To date, providing effective palliative care has become more and more difficult for specialists, as worldwide health systems become strained under the ongoing COVID-19 pandemic (16). Therefore, we have conducted this survey, from the professional perspective of medical workers, to explore the influence of COVID-19 on palliative care practice, and further put forward some interventions to deal with the status quo. Overall, this report not only identified specific aspects that have been negatively affected by COVID-19, but also underscored the need for useful coping strategies.

Although it is universally accepted that palliative care should be adopted by specialists in all oncology settings to benefit cancer patients and their families (17). The lack of integrating supportive care into comprehensive cancer treatment has become strikingly evident in the current context of the COVID-19 pandemic (18). In the present study, we found the majority of oncologists agreed that COVID-19 adversely impacted their routine clinical work (75.7%) and palliative care practice (64.9%) to a very large extent. Perhaps it is because this pandemic has created more uncertainty and disrupted the way that we practice medicine, including palliative and supportive cancer for cancer patients. Through ongoing international conversations pertaining to COVID-19, palliative care practitioners are asking whether we should attach more importance to patients with cancer, who are often the most vulnerable (19, 20). We consider that because of the underlying suppression of immune system and poor general condition heightening the risk for susceptibility to COVID-19 and relative complications, cancer patients ought to be paid more attention and supports (21–24). Another concern is that palliative care practice to address certain circumstances of cancer patients remains inadequate and immature, such as in the particular case of the severe COVID-19 pandemic (25). Despite the fact that individuals with cancer on active palliative healthcare are more likely to require frequent hospital visits or meeting with professionals, the isolation of interpersonal contact and restrictions on patient access to hospitals, in order to reduce the risk of spreading SARS-CoV-2, bring great difficulties to the practice and promotion of palliative care (26). Hence, palliative care in oncology should be an explicit part of international response plans for COVID-19, especially considering the high morbidity and mortality from COVID-19 in patients with cancer (27).

More importantly, 67.6% oncologists in our study considered that their personal lives and daily routines also had been significantly and negatively affected by the COVID-19 outbreak. In a study from Italy during COVID-19, frontline health care workers were reported severe posttraumatic stress symptoms, which could seriously affect their lives and careers (28). Previous studies also showed that medical workers tended to worry a lot about possibly infecting their families and thus usually were highly stressed even after coming back home (29, 30). Besides, numerous medical professionals around the world were sent to quarantine after contacting and fighting against COVID-19, which might cause a significant impact on the mental health and daily life of medical staff (31, 32). While patients are often the focus of attention during COVID-19, we believe that medical workers should be given full supports, appropriate comforts, and positive encouragements, as well, which may be one of the potential ways to motivate palliative care practitioners.

Furthermore, when it comes to the current status of the palliative care system in China, most specialists deemed that it remained underdeveloped. They also agreed that many other factors besides COVID-19 were associated with it, presumably on account of the unbalanced medical resources, conflicts of traditional values, and reluctance from patients (33–35). Thus, the development of palliative care still needs further efforts, not only in the current context of the pandemic but in the future. Despite challenges experienced during the pandemic, the global oncology community has responded with an unprecedented level of investigation and collaboration (36). This research also proposed some viable coping strategies, including promoting online palliative care guidance and home-based supportive care. Especially at present, the healthcare place is constantly shifting from the clinic to the home, where people can be treated *via* telehealth services, digital consultations, and intelligent devices (25, 37). These modern technologies are of great help and may encourage palliative care practice, even at the self-quarantine time during the COVID-19 outbreak. Similarly, based on digital equipment, remote multidisciplinary consultation and modern technology were also underlined by oncologists in this study. Emerging hi-technology will significantly contribute to palliative therapy if adopted properly and integrated into comprehensive care plan. However, novel technologies could augment traditional health strategies but cannot entirely replace them. As a result, shared decision-making, standardized palliative care training for medical workers, and personalized palliative medicine programs, etc., were also emphasized in this study.

Palliative care providers and hospice sectors play an essential role in the response to COVID-19 (38). Oncologists are known to provide supportive care with professional decisions, psychological counseling, and complex symptom management, especially for patients with advanced cancer (39). Providing such care is particularly challenging but also tremendously meaningful, given that humanitarian palliative caregivers with sufficient preparedness and capacity to cope with the current high-stress conditions of the COVID-19 pandemic may further improve the quality of life and optimize overall survival for cancer patients (35). Moreover, our study also highlighted that the issues and needs of palliative care practitioners should raise public concern and be further addressed.

There are several limitations. First, the sample size was moderate, but we have adopted some design strategies to improve response rates, including small financial incentives, mixed-mode survey, and brief questionnaire (40). In the current context of rapid spread of COVID-19, the opportunity to gather in-depth information was limited due to the extreme pressure on medical service system. Nonetheless, the results from our study supported a cross-sectional survey with larger sample to identify more examples of innovate practice in palliative care in the future. It also would be helpful to incorporate more diverse viewpoints from other palliative care participants in future research, such as nurses, as nurses are also involved in palliative care and COVID-19 to a large extent. Second, using self-reported results may bias the conclusion, although this study focused primarily on the subjective feelings of the oncologist community. Finally, the dynamic state of pandemic and different medical backgrounds among countries may limit the generalizability of the results for other settings. In the future, larger-scale studies involving more countries and regions to examine the impact of COVID-19 on palliative care under different epidemic prevention policies are warranted.

## Conclusion

The COVID-19 pandemic has a significant adverse impact on palliative care practice, daily clinical routine, and personal lives, from the perspective of oncologists. Most of them consider the current palliative care system underdeveloped in China, and other factors besides COVID-19 may be associated with this situation. The corresponding measures should be taken to improve the clinical practice of palliative care during COVID-19, such as incorporating more online guidance and remote assistive technology in palliative care, encouraging home-based and personalized palliative care treatment according to the condition of patients, and promoting up-to-date information and practical training for palliative care practitioners.

## Data availability statement

The datasets presented in this article are not readily available because questionnaire data and interview transcripts are available upon reasonable request. Requests to access the datasets should be directed to YW, m13992895327@163.com.

## Ethics statement

This study was approved by the Ethics Committee of National Cancer Center/Cancer Hospital, Chinese Academy of Medical Sciences and Peking Union Medical College. Informed consent was obtained from all individual participants included in this survey.

## Author contributions

YW, DW, and XM acquired, analyzed, and interpreted all data. YW and YH drafted and revised the manuscript. XZ and NB reviewed and edited this manuscript critically. All authors contributed to the concept and design of this work and approved the final version to be published.

## Conflict of interest

The authors declare that the research was conducted in the absence of any commercial or financial relationships that could be construed as a potential conflict of interest.

## Publisher's note

All claims expressed in this article are solely those of the authors and do not necessarily represent those of their affiliated organizations, or those of the publisher, the editors and the reviewers. Any product that may be evaluated in this article, or claim that may be made by its manufacturer, is not guaranteed or endorsed by the publisher.
